# Computer-controlled Intraligamentary local anaesthesia in extraction of mandibular primary molars: randomised controlled clinical trial

**DOI:** 10.1186/s12903-022-02194-2

**Published:** 2022-05-20

**Authors:** Rodaina H. Helmy, Sarah I. Zeitoun, Laila M. El-Habashy

**Affiliations:** Department of Pediatric Dentistry and Dental Public Health, Faculty of Dentistry, Alexandria University, Alexandria, Egypt

**Keywords:** Computer-controlled, Intraligamentary, Single tooth anesthesia, Local anaesthesia, Children

## Abstract

**Background:**

Local anesthesia (LA) poses a threat in children more than the treatment process itself, so pediatric dentists are always demanding less painful techniques. Computer-controlled Intraligamentary anaesthesia (CC-ILA) is designed to reduce injection pain and side effects of conventional techniques. The present study aims to assess the pain experience using Computer-controlled Intraligamentary anaesthesia (CC-ILA) during injection and its effectiveness in controlling pain during extraction of mandibular primary molars in pediatric patients.

**Methods:**

This randomized controlled clinical trial includes 50 healthy cooperative children, aged 5–7 years with mandibular primary molars indicated for extraction. They were randomly allocated to two groups according to LA technique: test group received CC-ILA and control group received Inferior alveolar nerve block (IANB). Pain was measured during injection and extraction: physiologically using Heart rate (HR), subjectively using Face-Pain-Scale (FPS), and objectively using Sound-Eye-Motor scale (SEM). Patients were recalled after 24-h to record lip-biting events. Data was collected and statistically analysed.

**Results:**

A total of 50 children (29 females and 21 males) with mean age 6.10 ± 0.76 participated in the study. There were significantly lower scores in the heart rate in the CC-ILA group during injection (*p* = 0.04), but no significant difference was recorded between the two groups during extraction (*p* = 0.17). The SEM and FPS showed significant lower scores in the CC-ILA group during injection (*p* < 0.0001, *p* < 0.0001) and extraction (*p* < 0.0001, *p* = 0.01) respectively. No children in CC-ILA group reported lip-biting after 24-h compared to 32% in IANB (*p* < 0.0001).

**Conclusion:**

CC-ILA provides significantly less painful injections than conventional techniques and has proved to be as effective as IANB during extraction of mandibular primary molars. An important advantage of this technique was the complete absence of any lip/cheek biting events.

*Trial registration* The study was prospectively registered in ClinicalTrials.gov with the identifier: NCT04739735 on 26th of January 2021, https://clinicaltrials.gov/ct2/show/NCT04739735.

## Background

Local anaesthesia (LA) forms the backbone of pain control in dentistry [1]. However, the injection poses a psychological threat especially in children due to the fear connected to needle puncture; which may even lead to complete avoidance and refusal to treatment [2]. Dental-phobia represents 5–15%. The strongest fears are associated with injections [3, 4]. Therefore, pediatric dentists are eagerly demanding for less painful techniques.

Historically, the Inferior alveolar nerve block (IANB) is the technique of choice for anaesthetising mandibular primary and permanent molars [5]. It affects a very wide area other than target teeth, so risk of cheeks and lip biting are major drawbacks of this technique in young children. Other rare but major complications include haematomas, risk of needle breakage, trismus and may even lead to transient facial paralysis [5].

In an attempt to overcome the limitations of the IANB technique, other methods have been advocated such as Intraligamentary Anaesthesia (ILA). This technique was introduced by Chompret [6]. Intraligamentary Anaesthesia is a method of intra osseous injection with LA reaching the cancellous space in the bone via the periodontal ligament (PDL), thus providing a rapid onset [7, 8]. This technique has superior features in cases with anatomical variations. It also prevents soft tissue injury, which is a major concern with others. However, it is believed that ILA has higher levels of post-operative pain than conventional techniques, and its duration only lasts for around 20 min [9–11].

Intraligamentary Anaesthesia can be delivered manually via conventional or high-pressure syringes [12], or delivered electronically using computer-controlled local anaesthetic delivery systems (CCLADS) [13]. There are some potential problems with the conventional intraligamentary technique such as the high pressure required to inject the solution, which may lead to breakage of the glass cartridge [14]. It could also cause trauma to the PDL tissues and extend the post-operative pain up to 30 days [15, 16].

The Wand-STA (Single Tooth Anesthesia) is the only CCLADS with incorporated dynamic pressure sensing (DPS) technology to monitor real-time pressure [17, 18]. Developed by Dr. Mark Hochman to reduce pain during injection, the STA-Intraligamentary Injection represents a new concept in LA techniques [19]. The system consists of a lightweight pen-like handpiece activated by a foot control which allows more precise LA delivery at a slow steady rate ahead of the needle with minimal tissue resistance [20]. One drawback in handling the STA system is that it may be complex for less trained dentists at first, not to mention that it is more expensive than a manual syringe [21].

The first study reported on Wand in children was by Asarch et al. [22]. The aim was to compare the effectiveness of CCLADS to the traditional syringe in restorative procedures. They reported no difference between both techniques, and general satisfaction of all participants. A systematic review and meta-analysis conducted by Libonati et al. [23] on 20 studies comparing CCLADS and conventional techniques concluded that CCLADS seem promising as it offers a less painful LA injection in adults and children; but more studies are needed to verify this. There is a lot of literature regarding CCLADS, but limited research is available on the use of CC-ILA with CCLADS on primary teeth [18, 24]. Recent clinical trials showed that CC-ILA reduced pain perception scores for primary tooth cavity preparation, pulpotomies as well as permanent teeth extractions [18, 25–33]. However, more studies need to evaluate effectiveness of CC-ILA in extraction of primary molars [24].

The present study aimed to compare the pain experience of CC-ILA and conventional injection by IANB and its effectiveness in controlling pain during extraction of mandibular primary molars in pediatric patients. The null hypothesis was that there will be no difference in the pain experience with the use of CC-ILA compared to the IANB in pediatric patients.

## Methods

### Study design

This study was a randomized controlled clinical trial that was conducted in the Pediatric Dentistry and Dental Public Health Department, Faculty of Dentistry, Alexandria University, Egypt. Participants were randomly allocated to two parallel groups according to the LA technique used: test group received CC-ILA and control group received conventional IANB. The allocation ratio was 1:1.

### Ethical consideration

The study protocol was approved by the Institutional Review Boards (IRB) of Research Ethics Committee, Faculty of Dentistry, Alexandria University, Egypt (IRB NO 00010556-IORG 0008839), and was prospectively registered in ClinicalTrials.gov with the identifier: NCT04739735. All procedures were performed in accordance with Helsinki Declaration and its later amendments. Reporting of the study follows the protocol established by the Consolidated Standards of Reporting Trials Statement (CONSORT) checklist [34].

### Sample size estimation

The sample size was calculated based on results obtained from previous studies of similar nature. It was estimated assuming alpha error = 5% and study power = 80%. Tekin et al. [35] reported mean ± standard deviation (SD) SEM score = 3.93 ± 1.223 when ILA was used, and 5.17 ± 1.891 when IANB was used. Based on comparison of means, sample size was calculated to be 25 per group, and the total sample size is 50 [36]. The sample size was calculated using powerandsamplesize.com calculator [37].

### Randomisation and allocation concealment

Participants were randomly allocated using a computer-generated list of random numbers. Block randomisation was used with random block sizes of 4. To ensure allocation concealment, each child was given a serial number written in identical sheets of paper with the group to which each child is allocated and placed inside opaque envelopes carrying their respective names. A trial independent personnel was assigned to the role of keeping the envelopes and unfolding them only at the time of intervention so that the group the child is allocated to was concealed from the outcome evaluator. Due to the nature of the study, the operator could not be blinded. However, the participants were blinded to the treatment groups, therefore, this clinical trial is single-blind.

### Eligibility criteria

Children aged 5–7 years old whom their mandibular primary molars were indicated for extraction were selected after thorough clinical and radiographic examination. These included non-restorable teeth due to primary or secondary caries, crown fractures, periapical disease and failed pulpotomies [38, 39]. History was taken to ensure subjects were free of any systemic disease or any known sensitivity to LA drugs. Participants were cooperative with Frankl behavioural rating scores 3 or 4 [40]. Teeth that showed any signs of mobility, acute pathosis, ankylosis, or root resorption affecting more than one third the root were excluded from the study. Parents of eligible children who agreed to give their consent were given detailed explanation of the purpose and methods of the planned clinical research including the benefits and risks of the study. Verbal and written informed consent for participation in the study was obtained from their parents.

### Intervention

The child’s first visit was a mean of introducing dentistry and acquainting the child to the dental unit and instruments using ‘Tell Show Do’ technique. No treatment was done in order to build a strong patient-dentist relationship.

(A) Injection with CC-ILA [18].

**Fig. 1:**
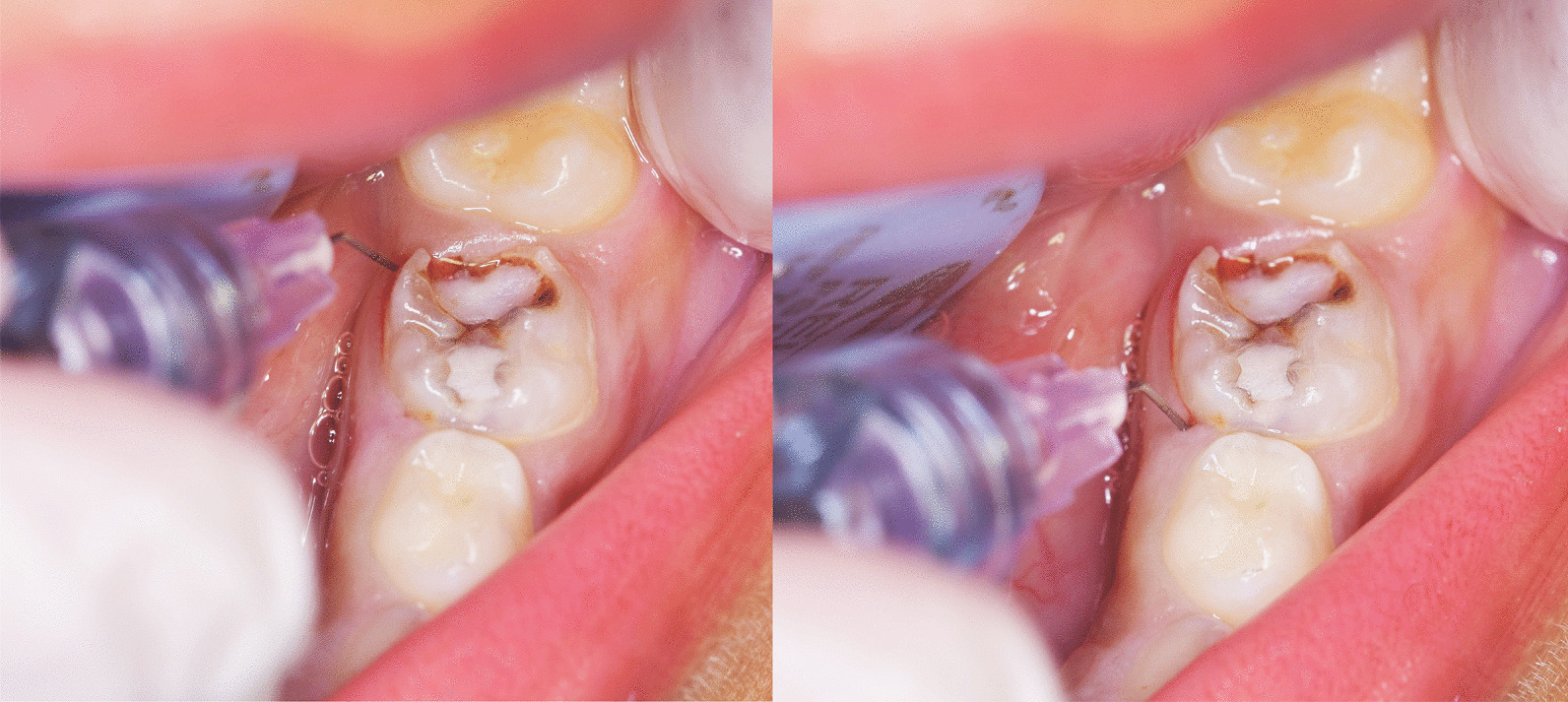
Administration of computer controlled Intraligamentary anaesthesia in a multi-rooted tooth. Left: first insertion on the disto-lingual line angle of the tooth. Right: second insertion on the mesio-lingual line angle of the tooth

During the intervention visit, soft tissues were dried, and 20% benzocaine topical anaesthetic gel (Iolite, Dharma Research Inc., USA) was placed at the injection site for 1 min. Computer-controlled ILA was administered to the test group using the Wand-STA system connected to 30-gauge ultra-short disposable handpiece (STA; Milestone Scientific, Inc., Livingston, NJ, USA). To improve the accessibility, the wand handpiece was shortened by breaking off a section of the handle. A standardised 1.8 mL LA cartridge of 4% Articaine hydrochloride with adrenaline 1:100,000 (ARTINIBSA, Inibsa Dental S.L.U, 08185 Lliçà de Vall, Barcelona, Spain) was placed. Participants were told that juice will be sprayed on their tooth using a tiny hose. The needle was bent 45° for proper placement and was directed into the gingival sulcus of the distolingual line angle of the target tooth at approximately 30° to the long axis of the tooth with the bevel facing the alveolar bone. Few drops of LA were deposited by activating the foot control, then the operator waited 5 s before needle was advanced into the PDL. More solution was administered until pressure was built on a special indicator. Injection was stopped by lightly tapping the foot control again. The same was repeated for the mesiolingual line angle. These are the most effective sites for multi-rooted mandibular teeth according to manufacturer instructions (Fig. 1) [18]. Approximately 0.2 mL of anaesthetic was deposited into the periodontal ligament of each root, so a total of 0.4 mL was administered per tooth [41]. Numbness was tested by placing a dental probe on the gingiva immediately and after each 5–10 s till full numbness was declared.

(B) Injection with IANB [5].

In the control group, a standard technique for Inferior alveolar nerve block was used supplemented with long buccal infiltration for the buccal gingiva. Soft tissues were dried, and topical anaesthetic was placed at the injection site for 1 min. A 27-gauge disposable dental needle (C-K Ject, CK Dental Ind. Co., LTD., Korea) was directed from the opposite side of the arch at the level of the occlusal plane until bony resistance was met. The needle was withdrawn 2 mm to aspirate, then 1.0 mL of Articaine hydrochloride 4% with adrenaline 1:100,000 was injected, followed by 0.5 mL as a long buccal infiltration distal to the second primary molar. Numbness was assessed by placing a dental probe on the gingiva after each 30 s as well as by checking for lower lip tingling.

### Extraction procedure

Extraction was accomplished in both groups according to AAPD guidelines [42]. Lower full crown forceps was used to apply slow continuous buccal and lingual force. Post-extraction instructions were given to participants. Planning for space maintenance was considered as well.

### Outcome assessment

**Fig. 2:**
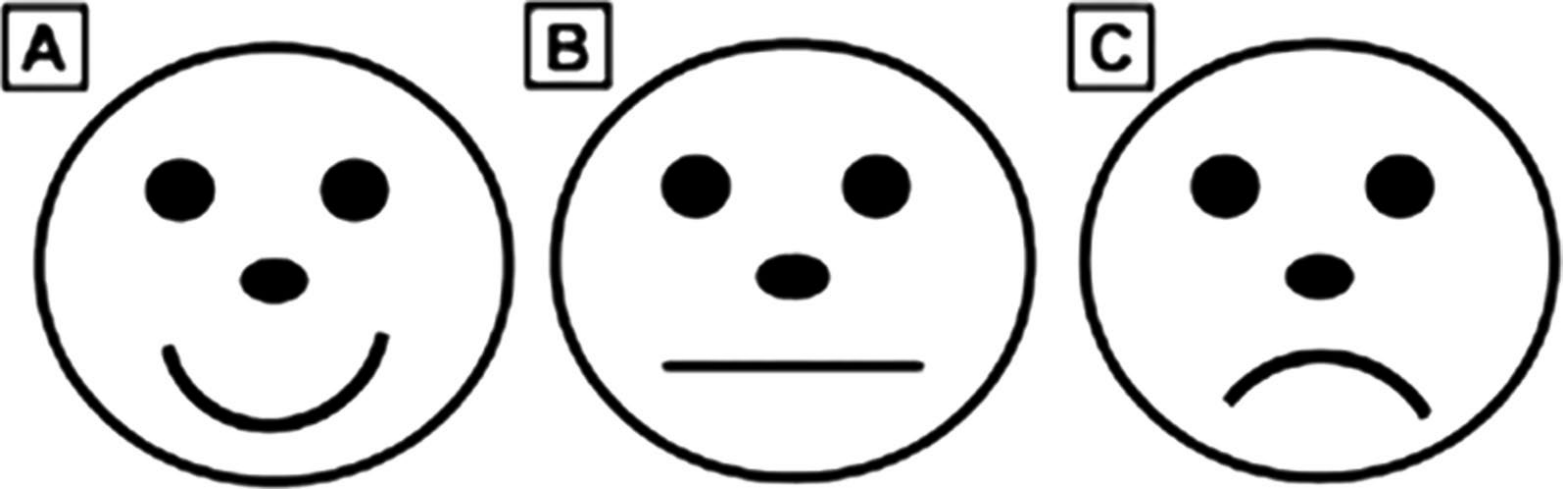
Faces pain scale (FPS) modified from the Maunuksela et al. [43] scale. **A** satisfaction; **B** indifference; and **C** dissatisfaction

(A) Pain was measured by three parameters:
1 Physiologically by recording Heart rate (HR) using a pulse oximeter placed on the child’s index finger at three time points: baseline, during needle insertion, and during extraction. Readings were recorded at 2-min intervals and average calculated.
2 Subjectively using a modified face pain scale (FPS) from Maunuksela et al. scale [43]. (Fig. 2) that comprises 3 faces with different expressions signifying: (a) satisfaction, (b) indifference, (c) dissatisfaction. Each child was asked to select the face that represented their experience right after injection and extraction.
3 Objectively using sound-eye-motor (SEM) scale (Table 1) that quantified the child’s pain response. Each of sounds, eye and body movements were graded from 1 to 4 during needle injection and extraction. It was evaluated using recorded video tapes and estimated by summing the three scores and calculating their average.
(B) Parents were recalled after 24-h following extraction. Recovery questions were asked to ascertain the occurrence of lip/cheek biting or any adverse events.

**Table 1:** Sound, eye, motor (SEM) scale

Parameter	Comfort	Mild discomfort	Moderate discomfort	Severe discomfort
Grade	1	2	3	4
Sound	No sound	Non-specific sound (probable pain)	Verbal complaint, louder sound	Verbal complaint shouting, crying
Eye	No sign	Dilated eye without tears (anxiety sign)	Tears, sudden eye movements	Crying, tears all over the face
Motor	Relaxed body and hand status	Muscular contraction, contraction of hands	Sudden body and hand movements	Hand movements for defense, turning the head to the opposite side

### Intra-examiner reliability

For standardization, all clinical procedures were performed by a single operator, who was trained and calibrated for the Wand-STA system. An impartial observer recorded SEM pain scores by observing and classifying each child’s behaviour on videotapes. After a 7-day interval, these steps were repeated to make sure the results were accurate and reliable. Intra-examiner reliability was tested by Intraclass correlation (ICC) [44]. The Intraclass Correlation coefficient yielded a score of 0.96, which ensured excellent agreement.

### Statistical analysis

Normality was checked for all quantitative variables using descriptive statistics, Q–Q plots, Histogram, and Shapiro–Wilk normality test. Heart rate measurements were normally distributed so means and SD were calculated. Mean HR measurements between both groups were compared using *T*-tests while intergroup comparisons were done using Repeated-measures ANOVA followed by post-hoc test with Bonferroni correction. For evaluating ordinal scales (SEM), median and interquartile ranges (IQR) were calculated. Mann–Whitney U tests were used to compare between the two groups. Wilcoxon signed-rank tests were used for comparisons within each group. Qualitative data were expressed as frequencies and percentages. Fisher’s exact test was used to assess post-operative lip biting. Monte Carlo simulation was used to assess FPS scale. All statistical analysis was performed with Statistical Package for Social Sciences (SPSS) software version 25. The significance level was set at *p* < *0.05*.

## Results

**Fig. 3:**
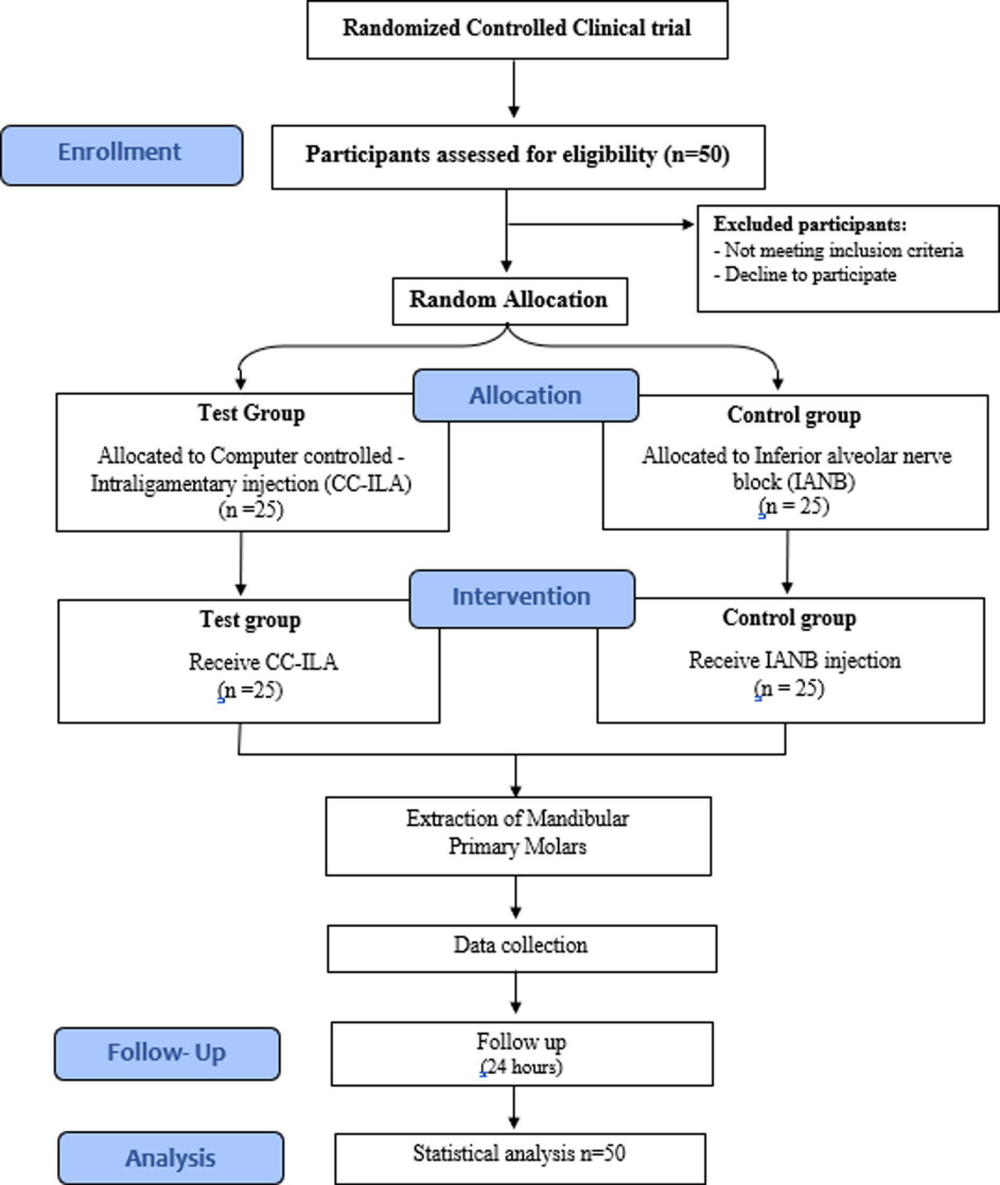
CONSORT flow chart study design

Subjects’ recruitment, allocation, intervention, and data analysis are illustrated in the CONSORT flow diagram (Fig. 3). A total of 50 children (29 females and 21 males) with mean age 6.10 ± 0.76 participated in the study: 25 in CC-ILA test group and 25 in IANB control group (Table 2). More first primary molars (60%) were extracted in the study than second primary molars (40%). No significant differences between the test and control groups concerning age (*p* = 0.36), gender (*p* = 0.77), or tooth location (*p* = 1.00). No failures were encountered with IANB in the present study; thus, no further injections were needed.

**Table 2:** Demographic characteristics of the participants (n = 50)

Variable	CC-ILA (Test group)	IANB (Control group)	Total	*P* value
Age (Mean ± SD)	6.20 ± 0.71	6.00 ± 0.82	6.10 ± 0.76	0.36
Gender N (%)				
Female	14 (56)	15 (60)	29 (58)	0.77
Male	11 (44)	10 (40)	21 (42)	
Tooth location N (%)				
First primary molar	15 (60)	15 (60)	30 (60)	1.00
Second primary molar	10 (40)	10 (40)	20 (40)	

*T*-test was used for age while X^2^ tests were used for gender and tooth number; SD: standard deviation

The baseline HR was not significant between both groups (*p* = 0.78) (Table 3). There was a statistically significant difference between the two groups in the mean HR during injection (*p* = 0.04), but not during extraction (*p* = 0.17). The mean HR measurements during injection in CC-ILA (104.64 ± 12.04) were significantly lower than IANB group (113.48 ± 16.66). Intergroup comparisons using Repeated-measures ANOVA followed by post-hoc test with Bonferroni correction revealed significant increase in mean HR from baseline to injection in each group (*p* < 0.0001, *p* < 0.0001), while no significant change from injection to extraction were noted.

**Table 3:** Mean Heart rate (HR) for the test and control groups

Heart rate (HR) (Mean ± SD) beats/min	CC-ILA (Test group)	IANB (Control group)	*P* value
Baseline HR (a)	99.92 ± 12.67	98.80 ± 15.30	0.78
HR during injection (b)	104.64 ± 12.04	113.48 ± 16.66	0.04*
HR during extraction (c)	107.68 ± 14.33	114.44 ± 19.57	0.17
Mean change in HR during injection (b–a)	4.72 ± 3.12	14.68 ± 8.91	< 0.0001*
*P* value^§^	*P* < 0.0001*	*P* < 0.0001*	
Mean change in HR during extraction (c–b)	3.04 ± 9.05	0.96 ± 11.97	0.49
P value^§^	*P* = 0.32	*P* = 1.00	
Mean change in HR from baseline to extraction (c–a)	7.76 ± 10.56	15.64 ± 9.53	0.01*
*P* value^§^	*P* = 0.004*	*P* < 0.0001*	

*Statistically significant at *P* value < 0.05

*T* tests were used to compare means; SD: standard deviation

^§^Repeated measure ANOVA was used with Bonferroni post hoc corrections for pairwise comparisons

Upon analysing pain by FPS, there was a statistically significant difference between groups in the satisfaction level after injection (*p* < 0.0001) and extraction (*p* < 0.0001). The FPS scores after injection in the CC-ILA group comprised 88% of the participants relating a satisfying experience while only a single child described dissatisfaction. In the IANB group, 56% satisfied patients were reported. After extraction, the percentage of satisfied subjects in the CC-ILA test group was 84%, compared to 52% in the IANB group (Table 4). There was no significance in comparing between FPS after injection and extraction within each group (test: *p* = 0.32, control: *p* = 0.66).

**Table 4:** Comparison of post-operative complications and face pain scales (FPS) between the test and control groups

Variable	CC-ILA (Test group)	IANB (Control group)	Total	*P* value
Lip biting after 24 h N (%)				
Yes	0 (0)	8 (32)	8 (16)	0.004*
No	25 (100)	17 (68)	42 (84)	
FPS after injection N (%)				
Satisfaction	22 (88)	14 (56)	36 (72)	0.001*
Indifference	2 (8)	0 (0)	2 (4)	
Dissatisfaction	1 (4)	11 (44)	12 (24)	
FPS after extraction N (%)				
Satisfaction	21 (84)	13 (52)	34 (68)	0.003*
Indifference	2 (8)	0 (0)	2 (4)	
Dissatisfaction	2 (8)	12 (48)	14 (28)	

Fisher’s exact test was used for lip biting, while Monte Carlo Simulation was used for FPS after injection and extraction

*Statistically significant at *P* value < 0.05

The SEM scales results showed a statistically significant difference during injection (*p* < 0.0001) and during extraction (*p* = 0.01) (Table 5). The mean SEM score in CC-ILA group during injection was 1.15 ± 0.27 and during extraction was 1.76 ± 0.95, which were significantly much lower than the IANB group (2.53 ± 0.88 and 2.53 ± 1.10 respectively). When comparing SEM scores from injection to extraction in each group; there was a significant difference in the test group (*p* = 0.01), but not in the control group (*p* = 0.97).

**Table 5:** Comparison between SEM scores during injection and extraction between both groups

Variable (Median (IQR)) (Mean ± SD)	CC-ILA (Test group)	IANB (Control group)	*P* value of MWU
SEM score during injection	1 (1, 1.33) 1.15 ± 0.27	3 (1.67, 3) 2.53 ± 0.88	< 0.0001*
SEM score during extraction	1.33 (1, 2.67) 1.76 ± 0.95	2.33 (1.67, 3.67) 2.53 ± 1.10	0.006*
*P* value^§^	0.01*	0.97	

MWU, Mann–Whitney *U* test; IQR, inter quartile range

*Statistically significant at *P* value < 0.05

^§^Wilcoxon signed-rank test

There was a statistically significant difference regarding the occurrence of lip biting after 24-h between the two groups (*p* < 0.0001). In the IANB group, 32% of the parents reported that their children suffered from lip biting issues, while no children in CC-ILA group reported any.

## Discussion

The results obtained from this study rejected the null hypothesis in which CC-ILA was found to be less painful than IANB during injection. Although CCLADS has proven to be effective, more research was needed to verify its effectiveness with CC-ILA in children [18, 24]. Most of the studies on CC-ILA compared it to maxillary buccal infiltrations [27, 30–33, 45, 46]; however, the present study added to the gap of knowledge where it is one of the few to compare between CC-ILA and IANB in extraction of mandibular primary molars. The IANB is the technique of choice for anaesthetizing mandibular teeth and is considered one of the most painful injections [5]. Additionally, dental treatment was limited to extractions because they induce very high levels of pain and stress and will thoroughly reflect the effectiveness of the LA technique. Parallel design was adopted in this clinical trial; the two groups were completely separated to avoid the negative effect of extraction on child’s behaviour in sequential visits.

It is always recommended to select at least two pain scales in conducting behavioural research specially in young children due to the limited cognitive, emotional, and social development compared to adults. Therefore, in the present study both subjective and objective scales were used [47]. Pain was measured subjectively by modifying the FPS from ElMaunuksela et al. to make it simpler to understand and interpret. This maximised the child’s response and decreased confusion. The SEM scale was used for objective assessment of pain as it has been used by many previous studies and has proved its accuracy to measure pain in children [18]. In addition to this, heart rate was recorded as it is one of the most accurate variables that reflect autonomic response to pain stimuli [48]. It omits the possible bias caused by the observer and the subjective self-reporting of the children.

The analysis of the acquired results showed that using CC-ILA in children resulted in significantly less pain experiences during injection than IANB measured by HR, SEM and FPS. This could be attributed to the slow controlled flow rate ahead of the needle which causes minimal trauma to the tissues [20], rendering the injection below the pain threshold of the child. Therefore, it potentially eliminates the painful experience. The pressure indicators allow precise LA delivery inside the PDL. On the other hand, the IANB technique is very traumatic as it requires a thicker and longer needle to reach deeper levels within the soft tissues.

These interpretations can also explain the results of the SEM intergroup comparisons. The pain encountered during injection of IANB elicited fear and negatively affected the child’s behaviour on the dental chair during extraction. Not to mention that the numbness felt can be a source of discomfort especially in the younger age groups. On the other hand, the very little pain experienced during CC-ILA injection enhanced the child’s cooperation, reaching the level of significance when compared to the pain encountered during extraction. This was not the case for IANB where no intergroup difference was detected. Tooth extraction is a very stressful procedure; the anxiety as well as the pressure felt during tooth movement could be expressed as pain. In the present study, it was notable that the mean scores recorded during injection and extraction in both groups were not extreme and did not include defensive movements.

The study results come in agreement with the studies conducted by Thoppe-Dhamodhara et al. [33], Garret-Bernardin et al. [27], and Patini et al. [31], who reported significantly lower mean HR using CC-ILA in maxilla compared to conventional buccal infiltration. Other studies by Baghlaf et al. [25] and Alamoudi et al. [26] compared CC-ILA to conventional IANB in children undergoing restorations and pulpotomies. Significant less pain scores with CC-ILA were reported. In 2019, Mittal et al. compared CC-ILA to conventional ILA in extraction of primary mandibular and maxillary teeth and confirmed the same results using FPS and SEM [18].

On the other hand, the acquired results were inconsistent with other two studies who reported that CCLADS and conventional techniques resulted in similar levels of pain perception in children; Versloot et al. [4] compared computer-controlled buccal infiltrations and CC-ILA to conventional infiltrations and IANB in the maxilla and mandible. They experimented a very wide age group (4–11 years), which included older children that are more capable of controlling their reactions, so the children might not have expressed pain although they could have felt it. In 2020, Smolarek et al. [45] conducted a systematic review and meta-analysis on 20 studies using CCLADS in children; only four studies used CC-ILA. The difference in results could be explained by the fact that there were many variables; they did not distinguish between different injection sites or techniques. It is well-known that LA injections in the maxilla are much less challenging with fewer anatomic variations compared to IANB, so the comparison may not have reached the level of significance.

The FPS and SEM showed significant differences between both groups during extraction, despite the fact that HR measurements did not reveal any significance. This could be justified by the child’s behaviour instead of the pain stimulus itself. It was noted in this study that CC-ILA showed more psychological acceptance by children than the conventional syringe as the handpiece-designed syringe resembled a pen and the ultra-short needle was easily hidden in the dentist’s hands which potentially reduces the stress and fear during injection. Moreover, children who received IANB encountered more pain during injection, which consequently affected their behaviour negatively. On the other hand, CC-ILA offered a less painful experience, which enhanced the child’s psychological behaviour, and overall satisfaction.

One of the most remarkable findings in this study was that no participants experienced lip or cheek biting issues in the CC-ILA group. This was consistent with the results of Giannetti et al. [28]. The CC-ILA affects only a single tooth without anaesthetizing the perioral tissues, which eliminates any cheek/lip biting events in comparison to IANB, which affects a very wide area other than target teeth, so children encounter total or partial loss of sensitivity of the lip and cheeks for the whole duration which may last for hours [5]. This temporarily hinders the child’s daily life with limited speech ability. No other adverse events were reported.

The use of CC-ILA combined the benefits of the potentially painless injection by providing needle-free experience using CCLADS, and the rapid onset of profound ILA achieved by delivering of solution directly into the PDL of the target tooth. It requires only a small volume of LA solution compared to larger volumes injected with IANB. Therefore, systemic toxicity is minimal since much less doses are being used [49]. Treatment started almost immediately; consequently, shorter dental visits were offered, and general satisfaction of the patients and their parents was noted in the present study. Not to mention that lip biting and numbness were eliminated post-operatively.

Putting all these advantages into consideration, CC-ILA can be a very practical alternative in children; especially if teeth in different quadrants are being treated in a single visit, which helps avoid management complications associated with multiple dental visits. Nevertheless, duration only lasts for 20 min [11]. This time was more than enough for teeth extractions, but further research is needed to evaluate its effectiveness using longer procedures, especially in uncooperative children and younger age groups to include it in routine pediatric dentistry.

Limitations to the present study may include the anxiety that often precedes any dental procedure especially extractions, as well as the pain experienced during injection, which may act as a confounding factor and affect the child’s behaviour on the dental chair. This could negatively impact the pain reaction measured by FPS and SEM scores during extraction.

## Conclusions

By analysing the results obtained, it can be concluded that CC-ILA provides less painful injections when compared to the conventional IANB and is more generally accepted by pediatric patients. It overcomes the side effects of other conventional techniques as it eliminates lip/cheek biting events as well.

## Data Availability

Not applicable. The dataset of the study can be made available by the corresponding author upon reasonable request. The data are not publicly available due to (restrictions, e.g., they contain information that could compromise the privacy of the research participants).

## Abbreviations

LA: Local anesthesia
ILA: Intraligamentary anaesthesia
PDL: Periodontal ligament
CCLADS: Computer-controlled local anaesthetic delivery systems
CC-ILA: Computer-controlled Intraligamentary anesthesia
IANB: Inferior alveolar nerve block
STA: Single tooth anesthesia
SEM: Sensory, Eye, Motor
DPS: Dynamic pressure sensing
CONSORT: Consolidated Standards of Reporting Trials Statement
HR: Heart rate
FPS: Face Pain Scale
SD: Standard deviation
SPSS: Statistical Package for Social Sciences
ICC: Intraclass correlation
IQR: Interquartile range
